# C-852-03. Lung *Prevotella Melaninogenica* Is Enriched Post Burn + Inhalation Injury, and Reprograms Innate Immune Cells In Vitro

**DOI:** 10.1093/jbcr/irag033.063

**Published:** 2026-04-06

**Authors:** Angel Charles, Robert Maile, Mariami Gogolishvili, Denise A Hernandez, Madelyn P Bucci, Shannon M Wallet, Ravinder Nagpal

**Affiliations:** University of Florida, Gainesville, FL; University of Florida, Gainesville, FL; University of Florida, West Palm Beach, FL; University of Florida, Gainesville, FL; University of Florida, Gainesville, FL; University of Florida, Gainesville, FL; Florida State University, Tallahassee, FL

## Abstract

**Introduction:**

The lung microbiome has been implicated in immune dysregulation after burn with inhalation injury. We investigated whether *Prevotella melaninogenica* (PM) is detectable in bronchoalveolar lavage (BAL), whether PM or its products alter innate immune responses in vitro, and whether BAL PM burden correlates with inflammatory readouts.

**Methods:**

BAL from patients (n = 12) with burn + inhalation injury underwent bacterial 16S rRNA microbiome profiling and targeted qPCR for a PM-specific genome sequence. In vitro, human monocyte, NK-cell, and neutrophil models were exposed to PM-conditioned broth (PMCB), with and without low-dose LPS stimulation, followed by multiplex cytokine assays and immune profiling for select cytokines and Damage-Associated Patterns (DAMPs). In a further cohort of patient BAL samples (n = 40), we enumerated PM-copy number using PM-specific qtPCR assay. Associations between PM copy number and other variables were tested using Spearman correlation with Benjamini-Hochberg false discovery rate (FDR) control.

**Results:**

Prevotella was among the most prevalent airway taxa by 16S rRNA profiling. PMCB reprogrammed immune pathways and altered cytokine secretion across monocytes, neutrophils, and NK cells, with a consistent significant additive effect on TNF, IL-10, and IL-6 secretion compared to PMCB-free controls. In BAL, the PM-specific qPCR signal was positively associated with multiple measures, including BAL cell-free DNA (q = 0.0019; n = 40), and IL-6 concentration (q = 0.0269), but not any other cytokine or other DAMP tested. These remained significant after adjustment for days post injury, sex, and TBSA.

**Conclusions:**

PM is prevalent in the injured airway, PM products reprogram innate immune cells in vitro, and BAL PM burden correlates with airway inflammation. These findings support a model in which PM contributes to maladaptive innate immune signaling after inhalation injury.

**Applicability of Research to Practice:**

Early identification of PM and targeted modulation of PM-driven pathways may enable risk stratification and immune-focused interventions to reduce pulmonary complications after burn with inhalation injury.

**Funding for the study:**

Funded by NIGMS and NHLBI.

